# An empirical evaluation of imputation accuracy for association statistics reveals increased type-I error rates in genome-wide associations

**DOI:** 10.1186/1471-2156-12-10

**Published:** 2011-01-20

**Authors:** Marcio AA Almeida, Paulo SL Oliveira, Tiago V Pereira, José E Krieger, Alexandre C Pereira

**Affiliations:** 1 Laboratory of Genetics and Molecular Cardiology, Heart Institute (InCor), Hospital das Clínicas, FMUSP- Universidade de São Paulo, São Paulo, Brazil

## Abstract

**Background:**

Genome wide association studies (GWAS) are becoming the approach of choice to identify genetic determinants of complex phenotypes and common diseases. The astonishing amount of generated data and the use of distinct genotyping platforms with variable genomic coverage are still analytical challenges. Imputation algorithms combine directly genotyped markers information with haplotypic structure for the population of interest for the inference of a badly genotyped or missing marker and are considered a near zero cost approach to allow the comparison and combination of data generated in different studies. Several reports stated that imputed markers have an overall acceptable accuracy but no published report has performed a pair wise comparison of imputed and empiric association statistics of a complete set of GWAS markers.

**Results:**

In this report we identified a total of 73 imputed markers that yielded a nominally statistically significant association at *P *< 10 ^-5 ^for type 2 Diabetes Mellitus and compared them with results obtained based on empirical allelic frequencies. Interestingly, despite their overall high correlation, association statistics based on imputed frequencies were discordant in 35 of the 73 (47%) associated markers, considerably inflating the type I error rate of imputed markers. We comprehensively tested several quality thresholds, the haplotypic structure underlying imputed markers and the use of flanking markers as predictors of inaccurate association statistics derived from imputed markers.

**Conclusions:**

Our results suggest that association statistics from imputed markers showing specific MAF (Minor Allele Frequencies) range, located in weak linkage disequilibrium blocks or strongly deviating from local patterns of association are prone to have inflated false positive association signals. The present study highlights the potential of imputation procedures and proposes simple procedures for selecting the best imputed markers for follow-up genotyping studies.

## Background

Genome-wide association studies (GWAS) are a promising tool for the identification of genetic markers underlying phenotypes of interest and recently allowed the identification of markers associated with several human complex phenotypes[1]. These studies have accomplished their goals in improving our knowledge of genetic patterns underlying diseases such as diabetes mellitus type I [1] and II [2] and Cronh's disease [3]. Although methodologically appealing, these high-throughput experiments are not free from biases and limitations. Indeed, it is highly acknowledged that GWAS are not only prone to major drawbacks such as genotyping errors and sample failures, but also to varying levels of genome coverage across samples [4]. In practice, a further complication arises from the barrier imposed by the comparison of results among different GWAS. The commercially available GWA platforms make use of distinct sets of markers with highly heterogeneous genomic coverage ranging from hundreds of thousands to millions of typed markers [5]. This diversity in panels of markers limits even further the full potential of genome-wide association studies to uncover variants putatively implicated in the susceptibility to diseases or other complex phenotypes of interest. This heterogeneity transforms the comparison, as well as, the combination of data results generated from distinct genome-wide panels into a challenging endeavor [6].

To overcome these issues, genotyping imputation algorithms were developed. These methods use information provided by high quality markers combined with genome structure information for the population of interest organized in the HapMap database. These procedures can potentially be a nearly zero-cost alternative to increase both power and coverage in individual GWA studies. The imputation procedures allow meta- and pooled analyses of GWAS data generated by distinct genotyping platforms, maximizing their overlap and, consequently, the number of typed individuals. Despite promising, the success of imputation algorithms are relative since they could also amplify non-detected technical errors in genotyped markers, the available HapMap information may not be well consolidated for the population of interest or the applied imputation algorithm may not be well suited for a specific dataset [7].

Here, we present a comprehensive comparative analysis of the data generated by the multipoint imputation algorithm and the data obtained by direct genotyping in a type-II diabetes GWAS dataset. This imputation algorithm uses a Markov chain to infer the allelic frequencies of a marker by the information provided by a large set of flanking markers. The analyzed dataset was generated and organized by the Welcome Trust Case Control Consortium (WTCCC) and is a constituent of a large epidemiological study focused in the determination of genetic markers that could predispose an individual to seven different diseases of interest [8]. In this scientific effort, a group of approximately 3000 healthy individuals was compared to groups composed by 2000 individuals accessed by diseases of interest such as: diabetes type-II, hypertension, coronary heart disease and bipolar disorder. These healthy individuals are part of two distinct cohorts selected to avoid population stratification, a very common source of bias in GWAS. Imputation algorithms currently available can use very distinct statistical approaches and, overall, their accuracy is satisfactory [3]. Details on the most recent methods, as well as their advantages and limitations, are reviewed and critically discussed elsewhere [9]. Our focus, in this report, is to describe how inferences based on imputed genotypes might impact the discovery of genetic markers possibly associated with complex phenotypes. The results presented here highlight the potential benefits and limitations of the use of imputed data in GWAS association studies for common phenotypes.

## Results

### Characteristics of the examined datasets

The results discussed herein are based on data available for approximately 2000 individuals accessed by type-II diabetes and 3000 healthy individuals (controls). We limited our evaluation to 387,662 biallelic markers with full information on both observed (genotyped) and imputed genotype frequencies. This set of markers covers all chromosomes, but sex-linked markers, having no major over-representation in any specific chromosome. A detailed list of the examined markers and their respective imputed and empiric frequencies is available upon request. In this report, the term empiric denotes markers whose allelic frequencies were determined by direct genotyping.

### SNP selection quality criteria

**Figure 1:**
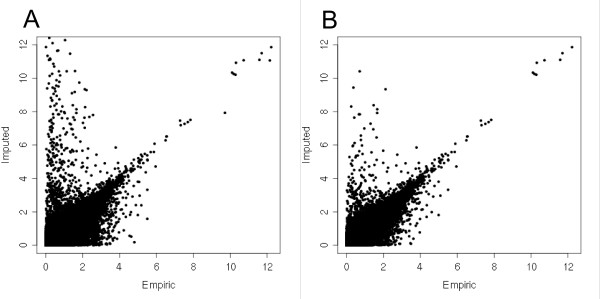
**Efficiency of filtering criteria**. Scatterplot comparing the minus-log corrected empiric and imputed p.values of the markers present in the complete dataset (A) and in the filtered one (B).

Association studies using empiric or imputed frequencies are very sensitive to low quality markers. Accuracy of the Impute algorithm is significantly reduced in alleles showing small MAFs or low calling posterior probabilities [4]. Initially, we determined the association statistics under different models of inheritance in the complete set of markers (See methods for further discussion). We applied a common combined filtering criteria composed of selecting only markers with calling probabilities higher than 0.95 and MAF (minor allele frequency) higher than 1%. To determine the efficacy of this procedure we used a dispersion plot (Figure 1) comparing the *P*-values (on a -log_10 _scale) assuming a multiplicative (log-additive) model of inheritance applied to empiric and imputed frequencies of markers in the complete and filtered dataset. The analysis of Figure 1 suggests that this standard quality control procedure was effective in excluding a group of 66.000 (17%) markers showing a significant difference in the magnitude of the association sign between empiric and imputed measures, these elements could potentially influence subsequent GWAS analysis. The same comparison was carried using the QQ plots graphical representation and, as expected, the use of this common filtering criterion was successful in reducing part of the spurious results (Additional file 1, Figure S1A). Both graphical representations highlight the effectiveness of quality and MAF filters for purging markers with low concordance between association statistics based on imputed and directly genotyped allelic frequencies. The term concordance used in this report refers to the discovery of markers showing evidence of association using a pre defined significance threshold, a concordant marker would be considered associated by their directly genotyped and imputed allelic frequencies. Nevertheless, several markers surpassing this quality control showed considerable bias between association statistics determined by the allelic frequencies.

### Imputed *versus *empirically genotyped markers: inflation of type-I error rates

Using the filtered dataset, the examined imputation algorithm, as previously described for allele and genotype imputation, also presents a high overall accuracy when comparing the association statistics generated using genotyped and imputed markers (Spearman's rank correlation coefficient, *r*_S _= 0.80). However, it is important to note some points. Firstly, the wide dispersion of dots around the upper left side of Figure 1 (panels A and B) indicates that, despite the overall good agreement, results that rely solely on imputed genotypes might reject more often the null hypothesis when it is in fact true. For example, at an α = 10^-5 ^we observed that inferences based on truly genotyped markers yielded evidence for 38 markers possibly associated with type-II diabetes, whereas this number would be 73 SNPs had the same inference been based on imputed markers solely. Approximately 47% of markers that would be considered associated by imputed allelic frequencies were discordant to the evidence derived from direct genotyping. Table 1 shows that similar results are also seen for different significance thresholds, suggesting that imputed frequencies might be more prone to false-positive associations independent of a specific pre-defined significance threshold.

**Table 1:** The use of different genomic significance thresholds and the number of SNP accepted as significant in empiric and imputed frequencies.

Sig. Threshold	Empiric	Imputed
10^-5^	38	73
10^-6^	16	43
10^-7^	13	33

To further analyse the nature of such type-I error inflation, we describe markers for whom their imputed association statistics were concordant or discordant in relation to their empiric association statistics. On Table 2 the results of such comparison highlight that there is a great concordance between association statistics determined by the allelic frequencies derived from imputed and genotyped information. A total of 317.255 markers were concordant, 317.217 were correctly considered not associated by both empiric and imputed frequencies and, a group composed by 38 markers was considered associated by both measures. Nevertheless, a group of 35 markers showed significant discordance between association statistics. The term discordance in this manuscript refers to differences regarding a pre-defined cut-off value observed in association statistics derived from imputed and directly genotyped allelic frequencies. Interestingly, in the diabetes dataset we found no situation in which an association would be claimed by empirically genotyped markers, but not by inferences relying upon imputed genotypes. The complete list of markers, association *P*-values and an indication of their concordance in terms of association statistics determined by imputed allelic frequencies can be obtained in Additional file 2, Table S1.

**Table 2:** Comparison of the number of markers that would be considered associated by empiric and imputes allelic frequencies

Number of markers		
Imputed	Empiric	
	p.value < 10^-5^	p.value >10^-5^
p.value < 10^-5^	38	35
p.value > 10^-5^	0	317182

To further explore the relationship between association statistics derived from imputed and from directly genotyped allelic frequencies, we sorted and determined the top ten markers that would be considered strongly associated based on the evidence provided solely by imputed frequencies (Table 3). This ranking analysis is extremely useful for the determination of markers strongly associated with a phenotype and avoids the error caused by an inadequate selection of a significance threshold. The analysis of the results organized in Table 3 highlight that association statistics for top-associated SNPs derived from imputed frequencies are highly inflated in comparison to their empiric counterparts. A huge proportion of these imputed markers (9 out of 10) would be considered not associated to the phenotype if evidence provided by direct genotyped allelic frequencies were to be used. It's an interesting fact that all these biased markers share similar frequencies in their minor alleles, all very close to 0,5, this allelic condition was further explored in this manuscript.

**Table 3:** Top associated markers based on imputed allelic frequencies

SNP	CHR	Position	Empiric	Imputed	MAF(emp)	MAF(imp)	STATUS
rs2000816	11	84151075	0,2382	1,57E-30	0,50	0,49	Discordant
rs4143896	14	41379353	0,4551	1,06E-19	0,50	0,49	Discordant
rs4982270	14	34950569	0,0853	5,72E-17	0,50	0,49	Discordant
rs10152907	15	52679008	0,3110	2,30E-15	0,50	0,50	Discordant
rs12900200	15	99971327	0,0394	2,80E-15	0,49	0,49	Discordant
rs35143	16	63543734	0,8364	3,62E-15	0,50	0,49	Discordant
rs2572406	8	11129662	0,0291	6,78E-15	0,49	0,48	Discordant
rs2996005	1	217884996	0,0010	5,61E-14	0,48	0,48	Discordant
rs696891	5	60940318	0,4430	9,85E-14	0,50	0,49	Discordant
rs4506565	10	114746031	6,02E-13	1,38E-12	0,35	0,35	Concordant

The column "Position" refers to the chromosomal position of the marker based on the reference genome (Hg18). The columns Empiric and Imputed organize the observed p values for the association with the diabetes phenotype based on directly genotyped and imputed allelic frequencies, respectively. The columns MAF(emp) and MAF(imp) refers to the minor allele frequency determined by allelic frequencies determined by direct genotyping (emp) and imputed (imp). The column STATUS describes if there is a concordance between association statistics based on allelic frequencies determined by both measures using a pre defined stringent significance threshold (10 -8).

The same analytic procedure was carried in polymorphic markers presented in the WTCCC hypertension dataset. Initially, we determined the set of markers from whom allelic frequencies were both directly genotyped and imputed using the haplotypic structure of flanking markers. The association statistics were generated and compared, similarly to the diabetes database approach, and similar results were also observed (Additional file 3, Table S2). Another important point is that, despite a considerably high correlation coefficient between association statistics, several hugely biased imputed markers could mislead follow up analyses. This finding appears rather contradictory but one should keep in mind that the correlation of minus log transformed association statistics is mainly defined by the immense number of markers showing good agreement between measures [9].

### Characteristics of the false-positive signals

Next, we sought to examine characteristics of false-positive associations that could be used as predictors of the quality of association signal derived from imputated markers. An analysis of the characteristics of false-positive signals is of paramount importance to guide investigators in appropriately evaluating *discovered *signals based on imputed markers. Here, *discovery *entails results crossing a specific α threshold under a frequentist perspective rather than a Bayesian approach. We selected an α = 10^-7 ^for illustrative purposes, an approximation that should work relatively well in typical studies conducted currently in Caucasian populations (CEU HapMap population, for example). Our empirical analysis demonstrated that the magnitude of the odds ratio of false-positive associations lies in the range of effects typically found in the GWA setting: median 1.26 (min = 1.20, max = 1.61); odds ratios were coined to be ≥1 for consistency. However, false-signals from imputed genotypes suggest more commonly protective effects (n = 47) rather than susceptibility effects (n = 26) for the minor allele variant.

**Figure 2:**
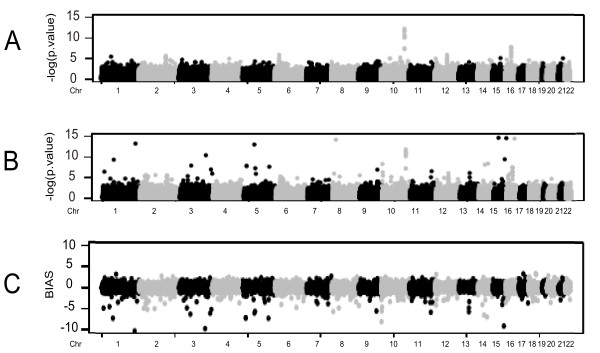
**Summary plots**. In Panel A: a graphical representation of the distribution of empiric association statistics throughout the human genome. In Panel B: same as A using the association statistics derived from imputed frequencies. In panel C: The distribution of the observed bias of association statistics of empiric and imputed frequencies.

It is accepted that some chromosomal regions, due to a higher number of recombination events, have less consolidated frequency panels in markers underlying these regions in human populations [4]. Allelic frequencies not well defined, or varying between populations, could significantly perturb inferences based on imputed markers in an association study. We investigated a possible relationship between specific chromosomal regions and false discovery events using imputed frequencies. The minus log transformed empiric and imputed *P*-values and the observed bias between associations statistics were plotted against their relative chromosomal positions (Figure 2) (See methods for further information). The term bias in this analysis refers to the algebraic difference between minus log transformed *P*-values determined by directly genotyping and imputation. The analysis of Figure 2c suggests the existence of genomic regions more prone to show major biases towards the alternative hypothesis of association when imputation methods were applied. Specifically, polymorphisms located at chromosomes 1, 3 and 15 showed the largest bias in favor of imputed measures. The most prominent biases are concentrated in imputed markers considered strongly associated (p < 10^-10^) to diabetes in contrast to their empiric frequencies, inflating considerably the number of associated markers. The number of these "low imputation quality" markers is limited especially when compared to the immense number of markers analyzed that could, consequently, pass undetected by common diagnostic analysis.

### Key indicators of a poor imputation performance on association statistics

Next, we carried out exploratory procedures to investigate key indicators of a poor imputation performance on association statistics. Specifically, we tested how the use of different quality calling criteria and the minor allele frequency (MAF) thresholds could predict the observed bias between empiric and imputed frequencies. This feature was explored by using more stringent cutoffs for calling rates, Hardy-Weinberg disequilibrium (HWD) and the use of SNPs showing a MAF ≥1%. The number of markers excluded by these quality filters was determined. The minus log transformed association statistics of the remaining imputed or genotyped markers were compared by analyzing their degree of correlation (Additional file 4, Table S3 and Additional file 5, Table S4). Consistent with findings from recent investigations studying the accuracy of imputation algorithms over genotype determination, the use of polymorphisms with a MAF below 1% accompanied or not by lower calling rates decreases the overall agreement between results based on imputed genotypes and those obtained by truly genotyped markers. In our examined dataset, HWD had no major impact on the performance of imputation since markers with strong Hardy-Weinberg deviation were already trimmed from the dataset before publication.

**Figure 3:**
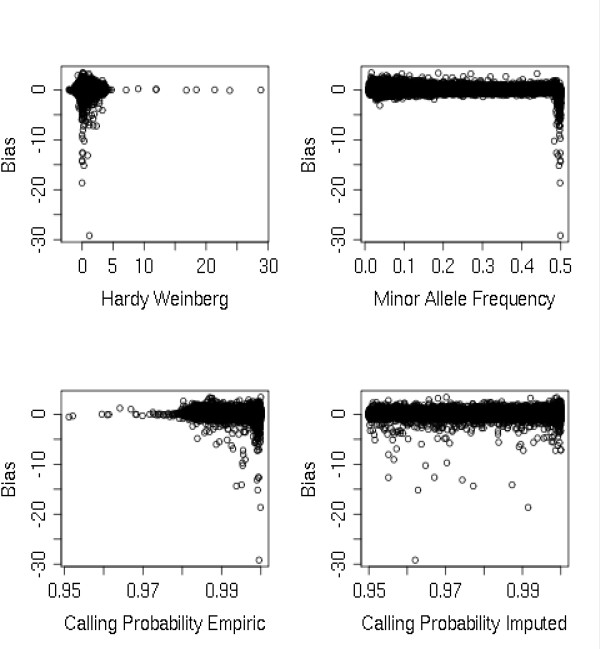
**Comparison of the predictive value of commonly used quality criteria for the observed bias between empiric and imputed allelic frequencies**. The minus log bias is plotted in the y axis and the tested variables in x axis.

To determine if the bias between association statistics could be predicted by common filtering criteria, we used a graphical representation plotting the observed bias between association statistics against calling probabilities, MAF and Hardy-Weinberg equilibrium of each marker in the dataset (Figure 3). The results of the plotted figure suggest that the Hardy Weinberg deviation, as expected, cannot be used as a predictive variable since the most prominent bias were encountered in markers that showed only a moderate deviation from equilibrium. The same procedure was applied to the empiric and imputed calling criteria, in both analysis the analyzed features do not show any predictive value, since the highly biased association values were concentrated in high quality empiric markers and randomly distributed for imputed markers. These features were useful for filtering highly biased markers in the creation of the filtered dataset but different thresholds of calling probabilities were not efficient predictors any further. When the same plotting routine was used using MAF as a predictor for bias (Figure 3), interesting results could be observed. Most prominent bias was encountered in markers with higher MAF (close to 0,5), suggesting that in this allelic condition the imputation algorithm was jeopardized by difficulties to determine the major and minor allele. This interesting feature was further analyzed to determine if this specific allele condition could be considered a useful predictor to identify these markers. We selected a subset of markers that were directly genotyped and imputed showing extreme MAF conditions (MAF < 0,01 and MAF > 0,49) and compared their transformed association measures with the use of dispersion plots and histograms (Additional file 6, Figure S2). The analysis of these figures show that the vast majority of markers in these allelic conditions have significant agreement for their association statistics but, as determined before, a limited number of markers, especially the ones showing MAF very close to 0,5, have an increased odds of being biased. The barrier is probably imposed by allele misspecification and is a challenging one, since a specific allele could be wrongly imputed leading to a totally spurious association statistic. A similar result was detected by an empirical evaluation of the imputation algorithm IMPUTE regarding accuracy of genotype determination [10].

**Figure 4:**
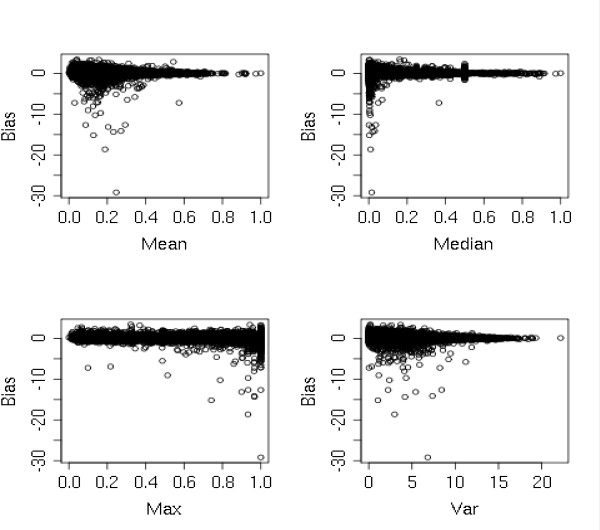
**Comparison of different r**^**2 **^**summary statistics of the complete set of haplotypic blocks and their use as predictive variables for the observed bias between empiric and imputed frequencies**.

The use of inconclusive or incomplete haplotypic information has long been considered a major source of errors in imputed frequencies, especially in chromosomal regions or populations with limited haplotypic knowledge. To test this hypothesis, we used r^2 ^SNP-by-SNP disequilibrium measure available in HapMap release (HapMap Public Release #22, 2007). Initially, we determined markers of our filtered dataset that were also evaluated in the HapMap database. This group is composed of 317,255 polymorphisms and it will be used in our further analysis. For each marker, a haplotypic block was determined using the complete set of markers showing a linkage disequilibrium measure to the specific marker (See methods for further details). Each haplotypic block is composed by a limited set of marker-to-marker r^2 ^statistics. In each block, four different descriptive statistics were evaluated: mean, median, maximum value and variance of the values of the r^2 ^statistic. We plotted separately the four statistics against the observed bias between association statistics of the complete set of markers (Figure 4). The analysis of the plotting organized in Figure 4 outlined that the use of the max value r^2 ^or the variance within a haplotypic block cannot be considered good predictors since the bias is randomly distributed throughout the variable values range. Otherwise, when the same procedure was applied to the mean and median values, significant insights could be obtained from the analysis. More prominent biases are concentrated in SNPs with relatively lower values, especially in the case of the median. Polymorphisms with lower mean or medians for r^2 ^measures have increased odds of showing higher deviation when the empiric and imputed association *P*-values are compared. Imputed markers showing these specific haplotypic conditions should be analyzed carefully before their use.

### Sliding window of association statistics

Finally, we explored the hypothesis that if a particular marker is truly associated with the investigated phenotype one would expect that close markers (in LD with the tested marker) would also present a higher chance of also being associated. In this scenario, a considerable proportion of markers flanking an associated SNP should also present significant levels of association for the phenotype under investigation. Imputed SNPs located in the same chromosomal region are inferred with similar accuracy since the same haplotypic structure information was used by the imputation algorithm. In the same hand, it is expected that a totally isolated associated marker within a well-known LD block will likely represent a false positive association. To evaluate the validity of this hypothesis, we developed an algorithm implementing a sliding window procedure that determines and collects minus log corrected association statistics of consecutive imputed markers using three different window sizes (1, 2 and 3 flanking markers) (see methods for further discussion). We determined different sliding windows size centered in the 73 imputed markers considered associated for diabetes II, these sliding windows were separated in two groups of sliding windows based if their central marker was concordant or discordant to empirically measured association statistics and three different summary statistics (mean, variance and total sum of corrected association statistics) were collected.

**Figure 5:**
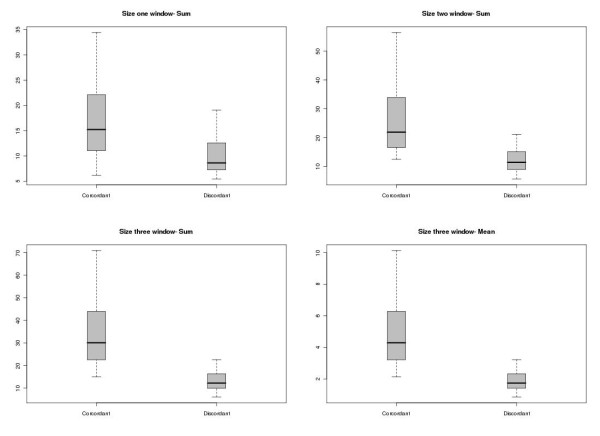
**Boxplot representation of the comparison of summary statistics of association values comparing sliding windows centered in concordant and discordant imputed markers**.

Using box plot graphical representations we evaluated the discriminative values of the different summary statistics of flanking markers as a predictor of imputation accuracy for association statistics. Interestingly, the use of the total sum of association values was very robust to efficiently separate true-positive and false-positive association statistics of imputed markers, independent of the size of the sliding window (Figure 5a-c). Using the mean value of the flanking association statistics was only useful in sliding windows of size three. Sliding windows centered in discordant markers (false-positive), as expected, showed consistently lower association statistics than sliding windows centered in true-positive markers. These results were especially impressive when using the total sum of corrected association statistics. The complete set of carried comparison can be inspected in Additional file 7, figure S3 and their application to WTCCC's hypertension results in Additional file 8, figure S4.

**Figure 6:**
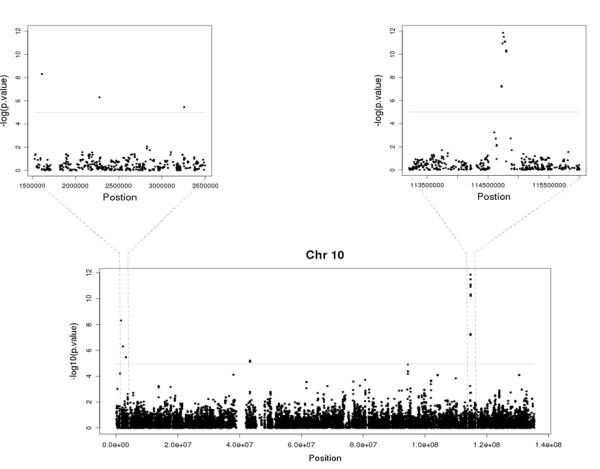
**Local patterns of association as predictor of accurate imputation**. On the lower graphic, a graphical representation highlights markers that could be considered associated to the phenotype under study using a significance threshold of 10 ^-5^. On the higher left and right panel, a highlighted representation of regions with concordant (right) and discordant (left) associations.

In a complementary way, we used a graphical representation to highlight this feature plotting the corrected association of statistics of sliding windows centered in true-positive and false-positive imputed markers considered associated in human chromosome 10 (Figure 6). In the upper left panel, a small region was highlighted showing three totally isolated imputed markers that could be considered associated to the phenotype of interest, but not by their empiric-derived statistic disfavoring the true/false positive ratio. When the same procedure was carried in imputed markers that were concordant to their empiric measures (upper right panel), it is noteworthy that a considerable proportion of markers weakly associated to the phenotype is also clustered in the same small chromosomal region. This result suggests that a preliminary analysis of surrounding markers could be used to flag and identify imputed markers that do not reflect the true empiric frequencies and could erroneously be considered associated.

## Discussion

Genome wide association studies are a promising tool for the determination of genetic signatures that could, when associated with environmental factors, predispose an individual to a phenotype of interest. Quality control of data in a GWAS study has been implicated as an important source of bias and loss of power in both linkage analyses and population-based association studies [6]. Imputation algorithms use allelic frequencies of typed markers and the haplotypic structure information to infer the expected allelic frequencies of a low quality or missing marker. These algorithms are considered a near zero cost alternative to allow the combination of results generated by different platforms with distinct genome coverage. The combination of directly genotyped and imputed allelic frequencies allowed the identification of SNPs that were strongly associated to diseases of interest such as hypertension and diabetes [1,8]. Genome wide association studies, like any other large scale experiments, are prone to false negative associations due to the impressive amount of hypothesis tests being performed and a small percentage of low quality SNPs can cause important statistical problems ([11]). These statistical limitations demand that any marker considered associated with a particular disease, specially imputed ones, should be directly genotyped using different genotyping platforms. This conservative procedure is now considered mandatory for the publication of such results. Nevertheless, even the follow-up of a small fraction of positive results from a GWAS involves significant costs.

The association of imputation procedures with low density chips can offer a convenient way to enhance the cost efficiency ratio and statistical power of a GWAS, since more individuals/markers can be typed by the same cost [12]. Several reports have compared the overall accuracy and statistical power of different imputation methods and highlighted the high genotype prediction accuracy of existing methods especially in genomic regions showing high LD (Linkage Disequilibrium) between markers[9]. Since imputation methods accuracy is closely related to the quality of the empiric frequencies used as an input, we initially determined the complete set of markers that were both directly genotyped and imputed by the multipoint imputation algorithm in WTCCC ([8]) diabetes II GWAS. This resulted in a set of 387,668 markers that were further evaluated. Using this set of markers, we tested a series of different quality criteria thresholds for MAF (minor allele frequencies), calling probabilities and Hardy Weinberg equilibrium deviation and analyzed the overall correlation between minus log transformed *P*-values of empiric and imputed allelic frequencies under a log-additive model of inheritance. We used a combined quality criteria of markers showing MAF > 0.01 and calling probability higher than 0,95 and filtered markers showing a considerably higher accuracy between association statistics using imputed and empiric frequencies (Figure 1). Using a minimum association threshold of 10 ^-5^, we identified a total of 73 imputed markers clamming association and among those, only 38 (52%) would be considered associated based on their empiric (directly genotyped) association statistics (i.e., nearly a half of imputed markers would be erroneously considered associated to the phenotype under study). The same pattern was observed when different and more stringent significance thresholds were used (10 ^-6 ^and 10 ^-7^).This result suggests that imputation methods are prone to inflate the number of markers considered associated in any of the evaluated thresholds in this report. These results are not a contradiction to the overall high accuracy for predicting genotype status previously described. These few highly deviated markers would be considered associated even when using highly stringent significance thresholds (< 10 ^-7 ^or lower) which could considerably jeopardize follow-up studies based only on association statistics of imputed markers. Since imputed markers are indispensable for merging the information generated by different platforms or studies (meta-analysis), it's important to identify these badly imputed and hugely biased markers.

In this report, we comprehensively tested several genotyping and imputation quality criteria, haplotypic information and chromosomal location as predictors of the quality of association statistics derived from imputed markers. As demonstrated in other reports dealing with the accuracy of genotypic determination, when the MAF of imputed markers are close to 50% [10] imputation accuracy greatly diminishes. We further analyzed a subset of markers that were selected based on their extreme minor allele frequencies (MAF > = 0, 49) to determine the validity of the evidence provided by this allelic condition for the identification of biased imputed markers. Indeed this allelic condition greatly predisposes imputed markers to have biased association statistics, but it can not be considered a good predictor since the majority of markers in this allelic condition show good agreement with directly genotyped ones in terms of association statistics. Imputed markers showing these specific allelic frequencies should be annotated and their use in follow-up studies should be done carefully. The other analyzed quality criteria, such as calling probabilities and Hardy-Weinberg Equilibrium deviation showed an even more limited use as predictors of false-positive associations derived from imputed allelic frequencies, since the bias between empiric and imputed association statistics was randomly distributed or clustered in markers showing high calling probabilities or very close to HW equilibrium.

A commonly accepted source of bias is the use of not well consolidated haplotypic information as an input for imputation algorithms. This could lead to imputed allelic frequencies not coherent to the population under study and, consequently strongly biased association tests. To explore this hypothesis we determined haplotypic blocks centered in each marker of the WTCCC dataset that were also present in the HapMap database. The comparison between the observed biases and four different summary statistics, representing haplotypic block consistency, showed a modest success when variance and maximum values were tested as predictors. Interestingly, the comparison between mean and median values of linkage disequilibrium as predictors showed that imputed markers located in regions showing weaker linkage disequilibrium structure are prone to higher bias. Their imputation and subsequent analysis under different genetic models of inheritance should be carefully done especially if the imputed marker is to be considered strongly associated to the phenotype under study. A similar result was suggested by Bakker P. I.W et al, when constructing a guide to the use of imputed information in meta-analysis of genome wide association studies[6].

The imputation algorithm overall accuracy for association statistics was compared and comprehensively evaluated under a diverse panel of different genetic conditions [13,3]. Here, it was showed that when allelic frequencies were imputed in markers located in low LD (linkage disequilibrium) regions, the accuracy of association statistics strongly diminishes. This restriction is probably imposed by the limited haplotypic information in these regions and to a not well consolidated haplotypic map. Based on the well known strong dependence between available haplotypic information quality and the accurate imputation of markers located in a specific haplotypic block, we developed an algorithm implementing a sliding window procedure focused in the analysis of association statistics of flanking markers as predictors for imputation quality of derived association statistics. Since the same haplotypic information is used for imputation of nearby markers it is expected that an imputed marker considered associated should be flanked by markers showing at least moderate association to the phenotype under study. Interestingly, imputed markers showing high concordance to empiric ones (for the derived association statistic) presented significantly higher total sum of association statistics as compared to false-positive markers. Indeed, the same procedure was applied to the complete set of imputed markers considered associated (10 ^-5^) in the WTCCC hypertension dataset with similar results (Additional file 8, Figure S4). The complex nature of WTCCC databases impose a barrier for the interpretation of results in this manuscript and could be considered a major source of the bias especially in imputed markers. This barrier originates from the fact that control and cases were not ideally matched in terms of their ancestry and it is expected that some association statistics derived from directly genotyped markers and especially from imputed markers are, indeed, susceptible to an increased odds of both type-I and type-II errors. Nevertheless, our results are concordant with the idea that additional information can be gathered from nearby markers in order to prioritize potentially associated markers for follow-up studies.

## Conclusions

Imputation algorithms are a convenient and low cost solution to increase the coverage and power of a performed GWAS, allowing comparison of already generated results and bridging the gap of distinct sets of markers in different GWAS platforms. Despite their, already evaluated, overall high accuracy for genotypic prediction, we describe that even after traditional filtering criteria, a considerable amount of markers may still present important problems when one is to evaluate the association statistics derived from these markers. We serially tested a group of features known as predictors for a low accurate genotype imputation. Mostly, these features were not able to robustly identify those markers from whom association statistics are significantly biased. One solution that seems to be robust is the use of information provided by flanking markers with the use of our sliding window procedure. It is expected that concordant imputed markers, showing agreement with association statistics derived from directly genotyped allelic frequencies, are located in haplotypic blocks composed by other markers showing, at least, a moderate association with the phenotype under study. Our results highlight the immense potential of imputation procedures, but are a reminder that indiscriminate use of imputed markers could alter the cost-effectiveness balance of follow-up genotyping efforts.

## Methods

### Determination of association statistics of a marker

The WTCCC consortium provided a complete panel of imputed and directly genotyped allelic frequencies of individuals accessed by diseases of interest and control individuals. The hypertension and diabetes datasets were downloaded and organized locally http://www.wtccc.org.uk/. Initially, we determined the complete set of markers that were both genotyped and imputed in each dataset. A Perl script was developed to generate a meta-population for each marker respecting the observed and imputed allelic frequencies for selected cases and controls. This script can be obtained by author request. Data were exported and analyzed by the specialized R package SnpAssoc [14], which determined for each SNP its association statistic under dominant, recessive, codominant and log-addictive models of genetic inheritance. This procedure was conducted, independently, by two co-authors (MAAA and TVP) and results were concordant. The complete set of association statistics was collected and organized locally and is available upon request.

### Analysis of specific chromosomal regions within studied markers

Markers typed in a specific chromosome were selected and sorted by their chromosomal position. The association statistics of a marker under a log-additive model of inheritance were collected and using a minus-log transformation plotted (Y-axis) with their position in the sorted vector. Markers in different specific chromosomes were plotted in grey and black, respectively. The bias was determined as the algebraic difference between minus log transformed association statistics derived from direct genotyping and imputation ((-log_10 _(*P*-value- empiric) - (log_10 _(*P*-value - imputed)). All the analyses were conducted on the R statistical environment; the complete set of developed programs can be obtained upon request.

### Determination of haplotypic blocks

HapMap haplotypic information was downloaded and organized locally (HapMap Public Release #22, 2007). A haplotypic block was defined as the complete set of r^2 ^marker-marker measures associated to a specific marker independently of the use of a pre defined minimum threshold for the r^2 ^measure. Once defined, these sets are informative for the determination of specific chromosomal regions under strong linkage disequilibrium. Each haplotypic block was characterized by their summary statistics and further explored for the identification of local patterns of strong association and the possible correlation between weak linkage disequilibrium regions and the accuracy of imputation derived association statistics.

### Sliding window algorithm

The complete set of minus log transformed *P*-values of imputed and directly genotyped markers under a log-addictive model of inheritance was collected and ordered based on chromosomal position. Imputed markers that were considered associated using a pre-defined threshold (10 ^-5^) were determined and classified into concordant and discordant markers in terms of their agreement within the association statistics. A locally developed Perl algorithm constructed sliding windows of different sizes (in this report 1,2 and 3) centered in the imputed markers (concordant or not) and collected the set of minus-log transformed association statistics of the flanking markers. A set of summary statistics, such as mean, median and variance of each sliding window was collected and referenced to the central marker. The complete set of raw results, summary statistics and markers comprised in each window can be obtained upon request.

## Authors' contributions

MAAA: Conducted the statistical analysis, the implementation of the computational routines and drafted the manuscript; PSOL: Supervised the implementation of computational routines; TVP: conducted data filtering analysis and statistical analysis JEK: helped the concept and design of the study. ACP: conceived the study and drafted the manuscript. All authors read and approved the final manuscript.

## Supplementary Material

Additional file 1 **QQ plots of entire and filtered datasets**. A. QQ plot of the entire dataset; B. QQ plot of dataset after standard filtering criteria. Click here for file

Additional file 2 **Complete set of imputed associated markers**. All markers that would be considered associated based on imputed allelic frequencies using a significance treshold of 10^-5^. A markers determined as concordant (STATUS column) could be considered associated by imputed and empiric allelic frequencies. Click here for file

Additional file 3 **Comparison of the number of considered associated markers by empiric and imputes allelic frequencies in hypertension database**. Determination of markers showing concordance and discordance of association statistics based on imputed and empiric allelic frequencies based on information provided by hypertension database of WTCCC. Click here for file

Additional file 4 **The effects of the use of different minimum thresholds for calling probabilities**. We serially tested a series of different minimum thresholds for empiric and imputed frequencies and analysed the effect of such filtering in the remaining dataset by determining the correlation of association statistics and the outlier percentage Click here for file

Additional file 5 **The effects of the use of different minimum thresholds for MAF (Minor allele frequencies)**. We serially tested a series of different minimum thresholds for minor allele frequencies and analysed the effect of such filtering in the remaining dataset by determining the correlation of association statistics and the outlier percentage. Click here for file

Additional file 6 **Dispersion plots and histogram of markers showing extreme MAF conditions**. Dispersion plots and histogram of markers showing extreme MAF (Minor Allele Frequency) conditions MAF < = 0,01 or MAF > = 0,49 Click here for file

Additional file 7 **Complete set of comparisons of different size sliding windows**. Each box-plot represents the tendency observed in association statistics of markers within different size sliding windows. Click here for file

Additional file 8 **Sliding window algorithm applied in hypertension dataset**. Each box-plot represents the tendency observed in association statistics of markers within different size sliding windows applied in hypertension dataset. Click here for file
