# Supplementary figures and images for: An empirical evaluation of imputation accuracy for association statistics reveals increased type-I error rates in genome-wide associations
Source: BMC Genet. 2011 Jan 20;12:10. doi: 10.1186/1471-2156-12-10 (PMC3224203; doi:10.1186/1471-2156-12-10)

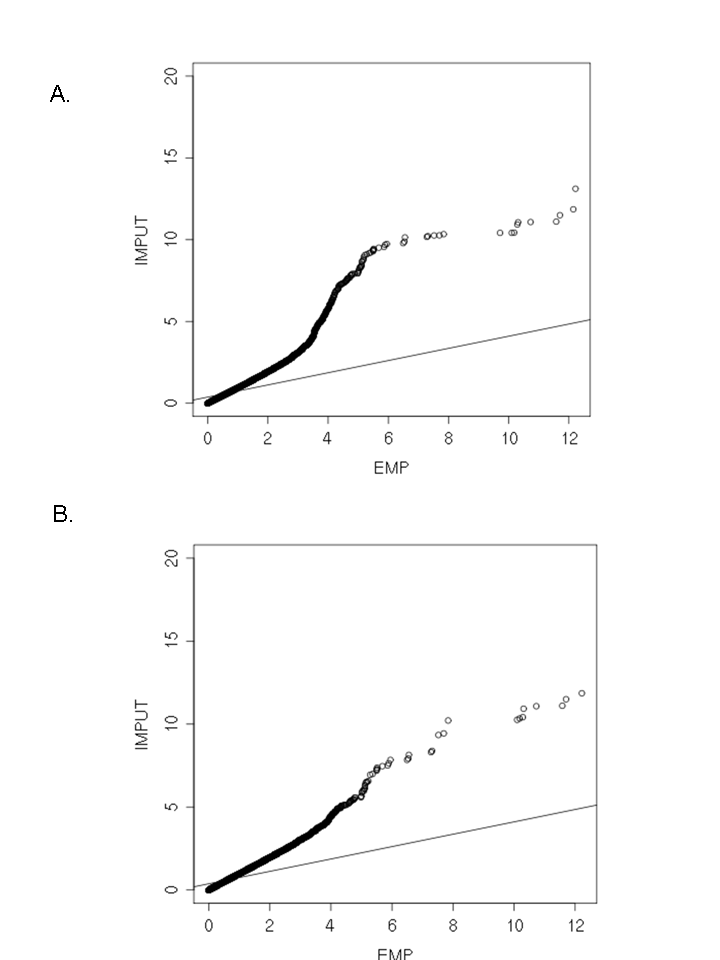

Supplement: Additional file 1 — QQ plots of entire and filtered datasets. A. QQ plot of the entire dataset; B. QQ plot of dataset after standard filtering criteria. [file 1471-2156-12-10-S1.PNG]

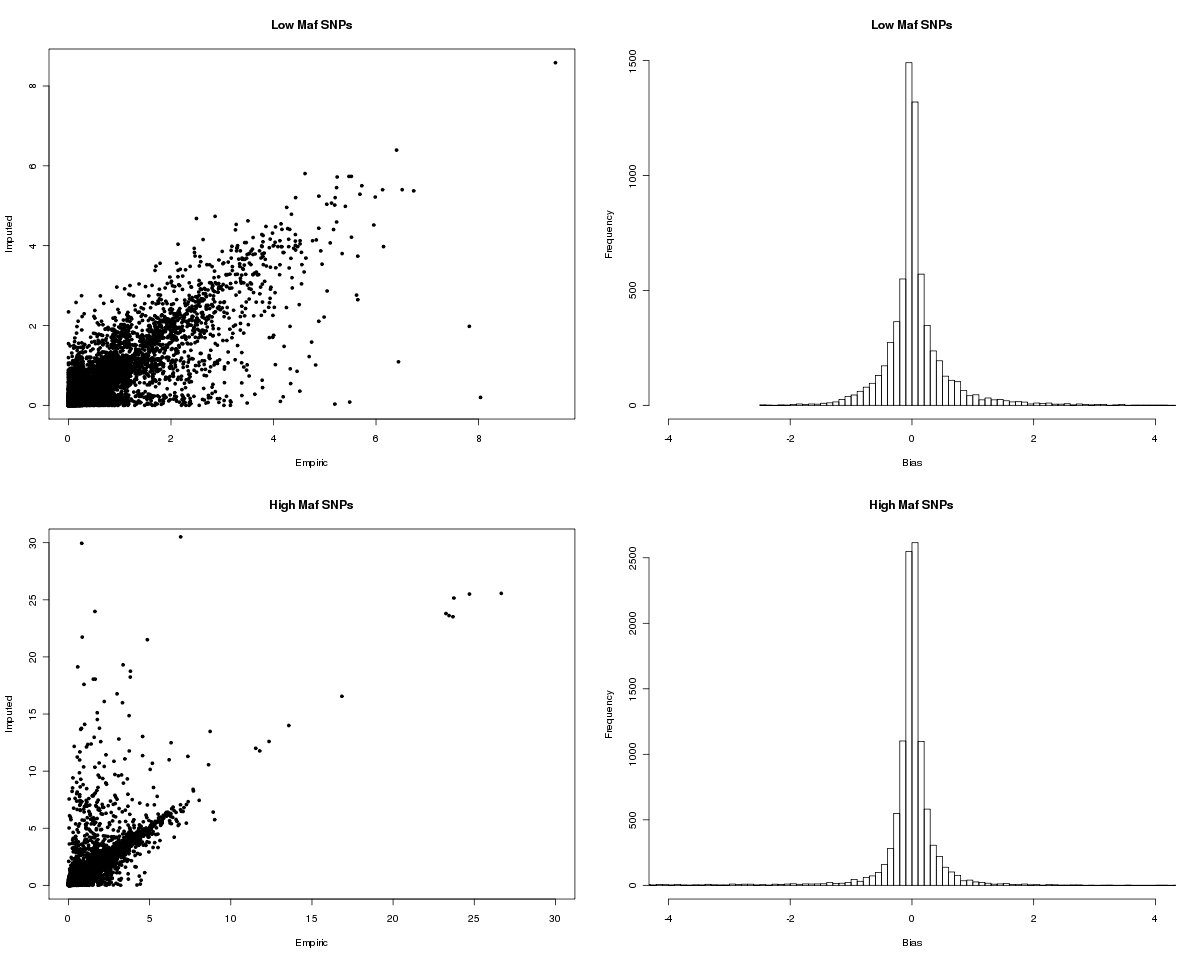

Supplement: Additional file 6 — Dispersion plots and histogram of markers showing extreme MAF conditions. Dispersion plots and histogram of markers showing extreme MAF (Minor Allele Frequency) conditions MAF < = 0,01 or MAF > = 0,49 [file 1471-2156-12-10-S6.PNG]

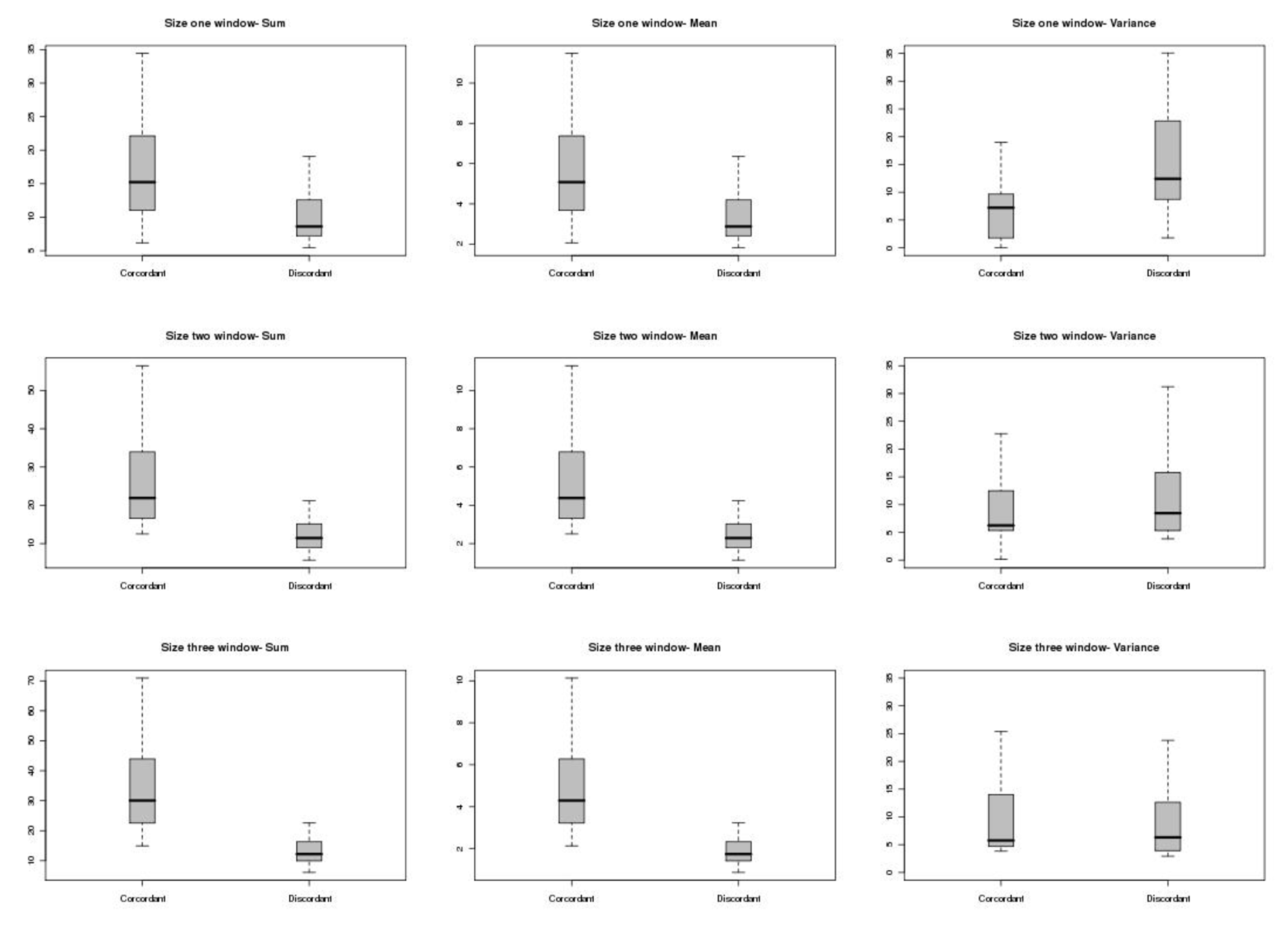

Supplement: Additional file 7 — Complete set of comparisons of different size sliding windows. Each box-plot represents the tendency observed in association statistics of markers within different size sliding windows. [file 1471-2156-12-10-S7.PNG]

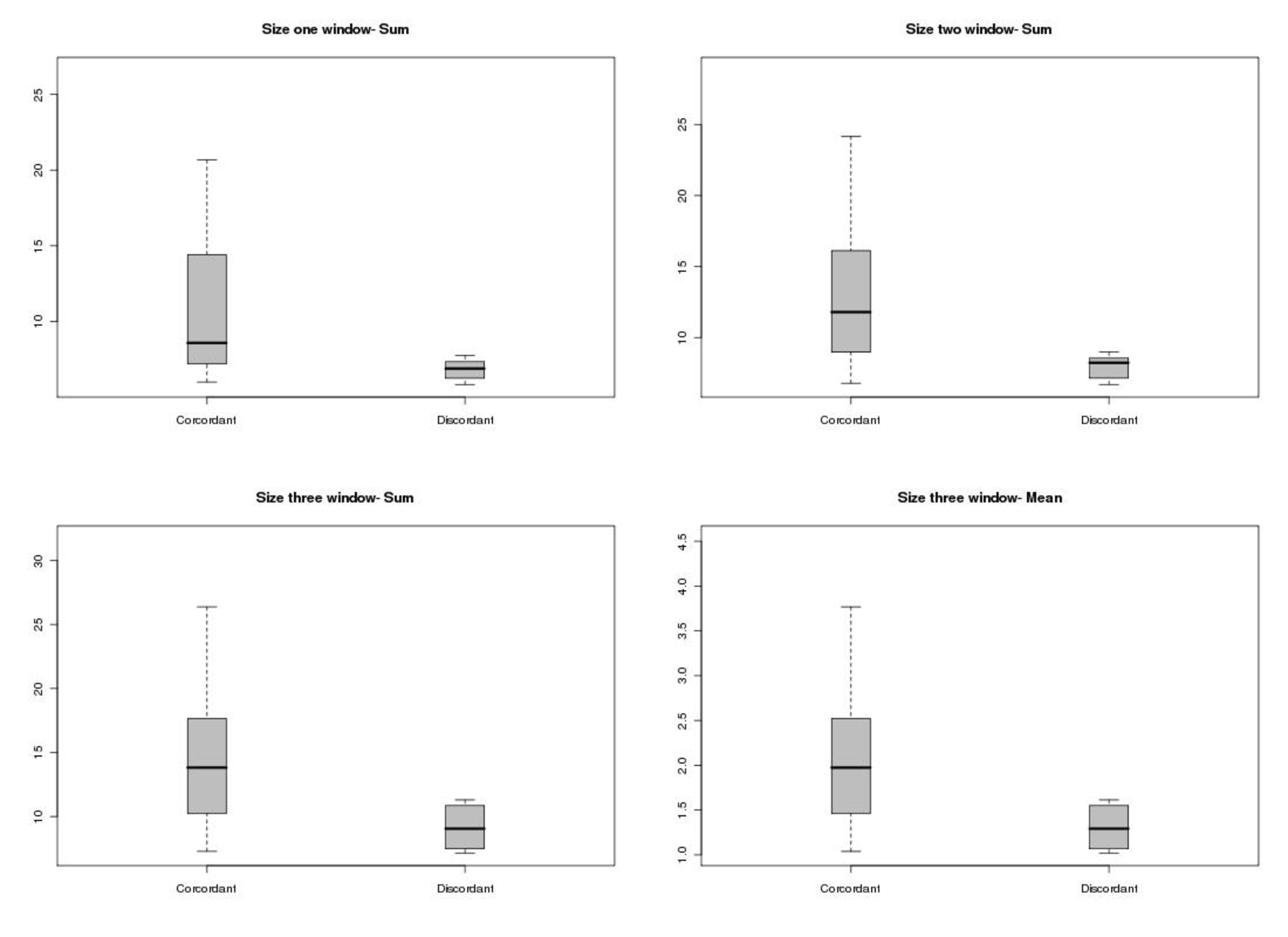

Supplement: Additional file 8 — Sliding window algorithm applied in hypertension dataset. Each box-plot represents the tendency observed in association statistics of markers within different size sliding windows applied in hypertension dataset. [file 1471-2156-12-10-S8.PNG]
